# Identification of climate induced optimal rice yield and vulnerable districts rankings of the Punjab, Pakistan

**DOI:** 10.1038/s41598-021-02691-4

**Published:** 2021-12-03

**Authors:** Azhar Ali Janjua, Muhammad Aslam, Naheed Sultana, Zia Batool

**Affiliations:** 1 Department of Economics, Government Graduate College, Hafizabad, Pakistan; 2 Department of Statistics, Faculty of Science, King Abdulaziz University, Jeddah, 21551 Saudi Arabia; 3 Department of Economics/Lahore Business School, The University of Lahore, Lahore, Pakistan; 4 Private Educational Institutions Regulatory Authority (PEIRA), Lahore, Pakistan

**Keywords:** Climate sciences, Environmental sciences, Environmental social sciences, Mathematics and computing

## Abstract

The study attracted to insinuate the inhabitant anomalies of the crop yield in the districts of the Punjab where climate variation, inputs utilization, and district exponents are indispensable factors. Impact evaluation of sowing and harvesting dates for rice yield has been analyzed. Suitable sowing and harvesting dates and potential districts for the crop are proposed. Data consisting of 13,617 observations of more than 90 factors encompassing valuable dimensions of the growth of the crops collected through comprehensive surveys conducted by the Agriculture Department of Punjab are formulated to incorporate in this study. The results establish the significant negative repercussions of climate variability while the impacts vary in the districts. The crop yield deteriorates considerably by delaying the sowing and harvesting times. Districts climate-induced vulnerability ranking revealed Layyah, Jhelum, Mianwali, Khanewal and Chinniot, the most vulnerable while Kasur, Gujrat, Mandi Bhauddin, Nankana Sahib and Hafizabad, the least vulnerable districts. Spatial mapping explains the geographical pattern of vulnerabilities and yield/monetary losses. The study ranks districts using climate-induced yield and monetary loss (222.30 thousand metric tons of rice which are equal to 27.79 billion PKR climatic losses in single rice season) and recommends: the formation of district policy to abate the adverse climate impact, utilization of suitable climate variation by adhering proper sowing and harvesting times, setting the prioritized districts facing climate-induced losses for urgent attention and preferable districts for rice crop.

## Introduction

Climate variability is an emergent issue that is hampering the life of human beings on earth whereas the foremost detrimental impact of climate variability has already been registered by various organizations on agriculture yield^1–8^. Global food production requires growth of about 70% till 2050, to nourish the increasing population^9^. However, climate change is likely to affect achieving sustainable food security especially in South Asia, where cereal yield is projected to decline up to 30 percent by 2050^4^. Rice is nourishing above 3 billion people universally and provides about 20% of humans’ total calorie intake^10^. In Asia, it is one of the main crops and has developed the backbone of the food security system^11^. It essentially requires a specific temperature as its growth gets highly influenced by changes in temperature^12^. As a second food crop, it is worst affected by climate change^13^. Kobata et al.^14^ recommended average optimal temperature for rice ranges from 23 to 29 °C through grain filling stage. During 2010–2017 the rice cultivation area declined about 10 percent with a reduction of 6.8 percent rice yield^15^ and is mainly associated with climate variation^16^.

Climate variability shows potentially inimical impact which depends on the warming rate, its duration, geographical region and vulnerability stage^17^. Agriculture-induced development, poverty alleviation, and food assurance are at stake. Shocks associated with climate variability are deteriorating rice yield which disturbing people’s affordability to consume nutritious food^18^. Vulnerability is the state of someone’s circumstances that make it susceptible to effect from natural or man-made hazards. It terrorizes the livings in developing economies where poor masses largely depend on the agricultural sector^5^ because of low adaptive capacities for proper coping mechanisms^19^. World Bank^20^ expects almost 40% to 80% of cropping land of Sub-Saharan Africa to drop till the 2030s to 2040s if the temperature warms from 1.5 to 2 °C, which is used to cultivate maize, millet and sorghum. Climate change may affect crop growth and yields through influencing crop-rotation and diversity^21^ vegetation dynamics^22^ agriculture and economy^23^ rice productivity^24^ risks of breadbasket failure^25^ food security^26^, the sensitivity of crop yield^27^ uncertainty of dairy systems^28^ constraints to the adaptive capacity of small farming^29^ economic value and risks^30^.

Pakistan may get adversely affected by climate change where the temperature is expected to increase up to 3 °C by 2040 and daily minimum temperature is becoming warmer compared to daily maximum temperature^31^. Pakistan is enlisted in the most vulnerable countries to climate change by Global Climate Risk Index^32^. IPCC (2018)^1^ estimated that failure to restrain an increase in temperature from 1.5 to 2 °C may trigger the yield decline of rice by rendering millions of people vulnerable. Agriculture is highly threatened and vulnerable to climate oscillations^2^.

GCRI (2009)^6^ ranked Pakistan 8th (7th) the country based on 1998 to 2017 (1996–2016) across the globe, which is adversely affected by climate change. Figure S1 depicts the temperature variability in Pakistan from 1960 to 2018. By 2050 Pakistan may face 19.5 billion dollars climate change loss from less output of wheat and rice crop^32, 33^, in a decade more than 80% of Pakistan’s, 124 districts and 33 million population faced natural disaster in some form and causing a 17.1 billion dollar loss to the economy^34^. GCRI (2009)^6^ out of 10 most climate affected countries from 1998 to 2017, eight were in the group of developing countries. Climate change is global in nature but worst in poor countries due to poor capacity to absorb change and less knowledge^35, 36^.

Shabir et al.^37^ investigated three districts on six transplanting dates. It projected an increase of mean temperature by 2.5 °C at Faisalabad, 2.9 °C at Gujranwala and 3.0 °C at Hafizabad and indicated the decline in rice yield. Further, the adaptation measures may increase the yield. Climate change impacts depend on the warming rate, its duration, geographical region and vulnerability stage^17^. Risks may be reduced by accelerating the transformational (changing the shape of inputs), cross sectoral (inter-related sectors of the economy i.e. more than one) and multi-level (different stages e.g. processing, creation, transmission, storage, social efforts, etc.) adaptations^1^.

The province Punjab produces over 60 percent of total rice and distinctive environment leads to produce 100 percent Basmati rice of the country^32, 33^. During 2008–2009 about 4.9 a million acres of land in Punjab is used for the cultivation of rice. Overall yield gap ratios to Punjab average are reported^38^ for rice as, Punjab average 1, Punjab progressive 1.8, Punjab potential is 2.1 and World’s best average is 2.2. Rice yield is less than its potential and climate variation is one of the main factors^16, 39, 40^. Shabir et al.^37^ pointed out the average temperature for rice growth is 20–25 °C and temperature beyond the upper limit reduce the grain size and weight by shortening the grain filling/development time duration^41^. Ahmad et al.^16, 39^ predicted a decline of 17 percent in rice yield due to an expecting increase of 2.8 °C in temperature. Anthesis rice stage is very sensitive and temperature above 38 °C even an hour may lead to negative repercussions^42^ while in districts of the Punjab temperature during rice phenology frequently cross it. However, adaptations are the key strategies to mitigate the negative consequences^43^ adjusting the sowing and harvesting dates^44^. Literature endorsed the regional specific adaptation strategies are more appropriate^37,45^. Abid et al.^46^ reported the impact of climate change across ecological zones of the Punjab and variation in the cropping calendar by exposing farming communities that are at higher risks. Naqvi et al.^47^ assert the need to identify regions and crops that are most vulnerable to climate variability so that an appropriate adaptation package may be initiated. This necessarily requires identifying the climate vulnerable districts for agricultural crops.

This study incorporates comprehensive quantitative and qualitative factors from all rice producing districts of Punjab. Investigating to find the impact of climate variation on rice yield from different rice sowing and harvesting time periods. Enlisting the district climate induced vulnerability, yield loss and monetary loss rankings for rice yield in Punjab and spatial mapping shows the geographical pattern of these rankings. Suggesting some adaptation measures to restrain the adverse climate impact on rice yield in the Punjab.

## Methodology

### Study area/case study

Pakistan stands among the top ten rice exporters and exports above 8% of global rice^48^. GCRI (2009)^6^ ranked Pakistan 8th country across the globe, which is adversely affected by climate change. During 2010–2017 the rice cultivation area declined about 10 percent with a reduction of 6.8 percent rice yield^15^ and is mainly associated with climate variation^16, 39^.

**Figure 1:**
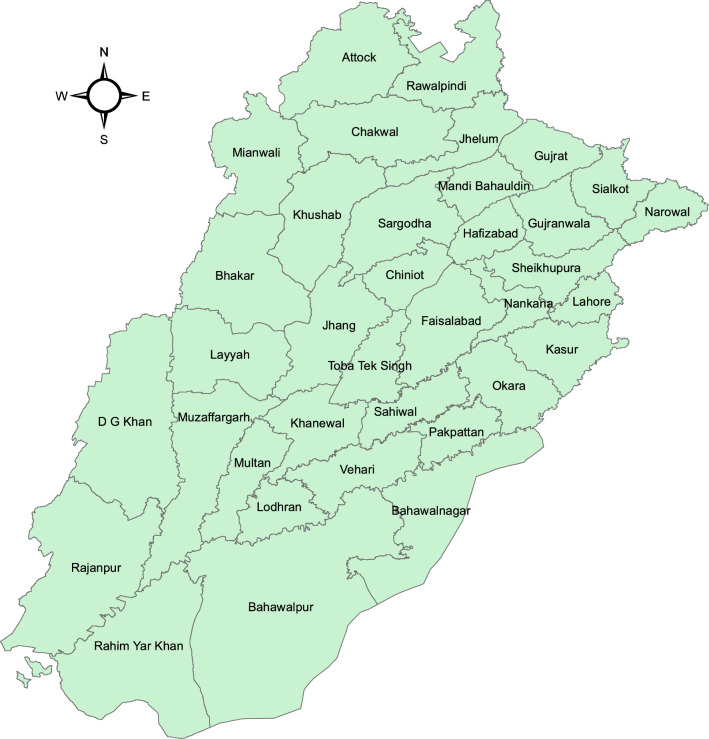
Map of province Punjab, Pakistan. *Source* Generating using Arc GIS version 10.3.1.

Figure 1 depicts districts of the province Punjab which produces over 60 percent of total rice and its distinctive environment leads to produce 100 percent of Basmati rice of the country^32^. Overall yield gap ratios to Punjab average are reported^38^ for rice as, Punjab average 1, Punjab progressive 1.8, Punjab potential is 2.1 and World’s best average is 2.2. Rice yield is less than its potential and climate variation is one of the main factors^16, 39, 40^.

### Sources of data

Every year agriculture department of Punjab holds field surveys in three rounds to estimate agriculture output of various crops subject to the availability of resources. The agriculture department of Punjab uses systematic random sampling, an overlapping sample of 1240 representative villages of the province where crop area (20 × 15 feet) from the representative village is the sampling unit.

### Data description

The dataset consists of 13,663 observations and after cleaning data by removing duplicate observations, outliers and missing data by using MS excel. The process confirmed the uniformity, completeness, accuracy, validity and consistency of 13,617 observations. Multiple inputs are used during crops growth thus the combination of input factors consists of dichotomous, polytomous and discrete variables. Variables with “k” multiple dummies are coded which makes the dataset a comprehensive combination of input factors utilized for crops growth.

Table 1 shows value of sowing time ranges from 1 to 6. Each numeric digit represents a separate sowing time period. Sowing time starts from May to August onward and is divided in six time periods, similarly harvesting time ranges from 1 to 5 (the detail is given below). This investigation includes 16 rice varieties; seed consist of three types and similarly others categorical variables each with subcategories can be traced in Table 1. Rice is cultivated in 32 districts of the province Punjab out of 36 districts. The variables carry 13,617 observations and almost similar mean and median values can be traced which shows the normality of data.

**Table 1:** Summary statistics of rice variables.

Variables	N	Mean	Median	Skewness	Kurtosis	Max.	C.V	Min.
Yield	13,617	11.0929	11.1	0.0204	3.6968	25.2	0.2681	0
Sowing time	13,617	2.9018	3	0.7646	2.8472	6	0.4123	1
Harvest. time	13,617	2.4978	3	0.1360	1.3653	5	0.5756	1
Rice varieties	13,617	7.2384	6	1.1533	2.9860	16	0.5442	1
Seed type	13,617	1.9571	2	0.0668	1.8640	3	0.3744	1
Seed quantity	13,617	5.0825	5	− 0.6824	2.7477	6	0.1774	2
Soil type	13,617	1.3676	1	2.2768	6.4688	4	0.6726	1
Plough	13,617	2.8143	3	− 0.1054	1.9462	4	0.3266	1
Sow. mode	13,617	1.3701	1	0.5383	1.2898	2	0.3524	1
Cutting mac.	13,617	2.1206	2	− 0.2373	1.3185	3	0.4173	1
Dap	13,617	38.8540	50	− 0.4057	2.9200	150	0.6173	0
Urea	13,617	74.9835	75	0.4345	3.2313	200	0.4072	0
Watering no.	13,617	19.8871	19	0.2107	2.4210	43	0.3137	1
Irrig. mode	13,617	2.3206	2	− 0.2769	2.3551	3	0.2592	1
Attack pest	13,617	1.6980	2	− 0.8626	1.7441	2	0.2704	1
Attack weed	13,617	1.7013	2	− 0.8794	1.7734	2	0.2691	1
Spray ps. no.	13,617	1.2688	1	0.9163	4.7896	11	0.7804	0
Last crop	13,617	5.3097	6	− 1.8107	4.6019	6	0.2766	1
District id	13,617	16.6061	16	0.1714	2.3553	32	0.4691	1

*N* number of observations, *Mean* arithmetic mean, *Max* maximum, *Min* minimum, *CV* coefficient of variation.

**Figure 2:**
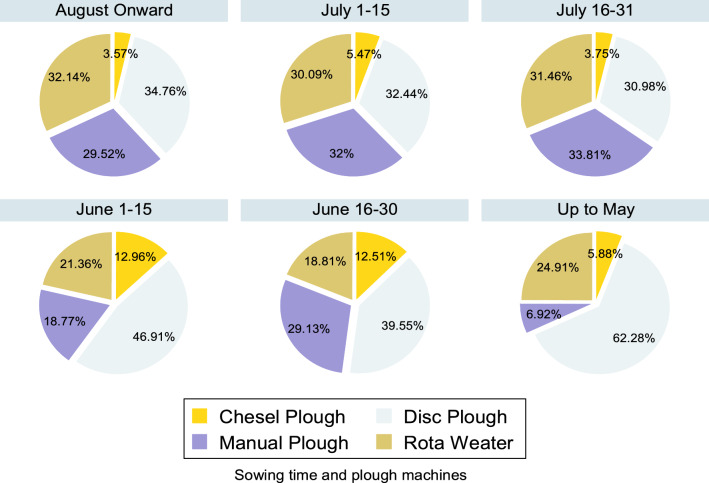
Sowing time and plough machines used for rice growth.

Figure 2 depicts the usage of types of machines for plough with a sowing time period for rice growth. Considerable variation in usage of plough machines can be seen however it shows disc plough is widely used irrespective of sowing time.

**Figure 3:**
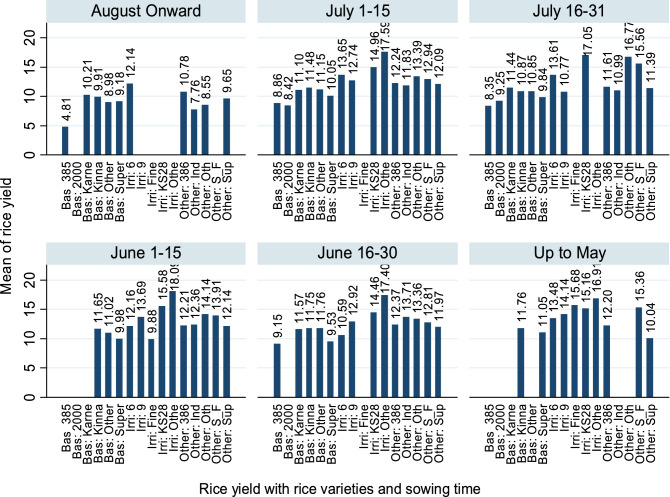
Rice varieties with mean of yield and sowing time.

Figure 3 shows the rice yield with rice varieties and sowing time. The yield differentiation of the same rice variety is evident of effects by variation in sowing and harvesting times, including other factors as well. Many factors may change the farmers’ decision which causing variation in sowing or harvesting may include e.g. availability of finance for seed, crops rotation, land agreements, the practice of farmers, instructions of concerned organizations, etc. Table 1 show variables included in this study. Sowing from August onward shows a clear reduction in rice yield; however, the good yield can be seen against sowing during June and July.

Table 2 shows the distribution of sowing time and cutting time which explains that more than 70% of sowing was completed in July and 44% of harvesting was done in the first half of November. The season of sowing and harvesting (each) constitutes about 3 months however the climate variation patterned in Fig. S1 which varies monthly and in the annual season as well. The changing climate urges use to investigate the yield optimal season for the crops.

**Table 2:** Frequency distribution of categorical variables for rice.

Variables	Frequency	Percent	Cumulative freq.
**Sowing time**			
Up to may	289	2.12	2.12
June 1–15	810	5.95	8.07
June 16–30	1919	14.09	22.16
July 1–15	5172	37.98	60.14
July 16–31	4587	33.69	93.83
August onward	840	6.17	100
Total	13,617	100	
**Cut date**			
Up to Sep.	694	5.1	5.1
Oct. 1–15	2097	15.4	20.5
Oct. 16–31	4295	31.54	52.04
Nov. 1–15	5991	44	96.03
Nov 16–30	540	3.97	100.00
Total	13,617	100	

### Theoretical framework and estimation

Endogenous growth theory^49^ asserts growth phenomenon determined with adoption of domestic policies while the intensive growth asserts the efficient utilization of inputs. Workers in agriculture may earn more wages due to increased output and at the same time food prices may decrease with stable supply. It leads to reduce the proportional income spent on food and ultimately increases the real income. Recent literature witnessed the gravity of the reputation of input utilization^50–54^ and the availability of a handsome amount of literature witnessed the impact of climate change on crops^55–65^. Tu et al.^54^ experimented on rice plant growth after twenty days of sowing and suggested the adjustment of sowing days to avail the suitable temperature which stabilizes and improves rice grain. Abbas and Mayo^66^ studied the impact of temperature on rice production during growth phenology and concludes that increasing temperature during replantation stage negatively affects rice plant by reducing the number of plants. Climate variation declines crop output through growth mismatching climatic factors^47, 67–69^.

A decline in crop production, due to climate change not only affects the agricultural sector, but its results may extend to all agriculture related industries and beyond, such as manufacturing and services. The change in crop production will have a multiplier effect. Climate-led changes in crop production will have consequences on the returns of factors of production, incomes and households’ consumption. A huge rise in commodity prices will create a great challenge for livelihood^32, 33^. predicted price an upward pressure in rice price by 31 percent and the resulting enormous increase in commodity prices will pose significant challenges for the country’s livelihoods.

Keeping in view the recommendation of available literature this study investigates, the climatic impressions that are creating anomalies in rice yield, to safeguard human life through adaptations measure(s) to enhance yield. This study internalizes the climate variability to investigate for the yield optimal sowing and harvesting time periods in Punjab. An attempt is initiated to suggest the ranking of districts based on loss is being occurred there due to climate variation for further policy insinuation.

Regression analysis treats nominal factors using dichotomous or polytomous variables along witn quantitative counterpart(s) which may helpful for this investigation. For the purpose, functional form of the variables may be given as.

Yield is a function of climate variation

1 $$Y = \, f\left( {CL} \right).$$

Impact of climate variation is captured by divided it into sowing and harvesting seasons

2 $$Y = f\left( {SO,HR} \right).$$

To capture the impact of climate variation efficiently sowing and harvesting seasons are further bifurcated in intervals. Sowing and harvesting seasons are divided into six and five intervals, respectively. This may be written as

3 $$Y = f\left( {SO_{i} , \, HR_{j} } \right),$$

where *i* = 1, 2, 3, 4, 5, 6 and *j* = 1, 2, 3, 4, 5.

Each interval may capture the impact of climate variation during these specific sowing and harvesting days.

4 $$Y = f\left( {\mathop \sum \limits_{i = 1}^{6} SO_{i} ,\,\mathop \sum \limits_{j = 1}^{5} HR_{j} } \right).$$

Yield as a function of crop sowing and harvesting intervals with the inclusion of other input factors which may make the functional form of the model as

5 $$Y = f\left( {\mathop \sum \limits_{i = 1}^{6} SO_{i} ,\,\mathop \sum \limits_{j = 1}^{5} HR_{j} ,\,\mathop \sum \limits_{w = 1}^{n} OT_{w} } \right),$$

where *SO* Sowing time (*i* = 1, 2, 3, 4, 5, 6), *HR* harvesting time (*j* = 1, 2, 3, 4, 5), *OT* other factors (*w* = 1, 2, 3,….*n*) and *Y* rice yield.

Estimation of a model using regression analysis requires the specification of variables in equational form. Rice cultivates in 32 districts and each district may carry specific characteristics for rice yield. To capture the district impact inclusion of district variable may form the econometric model as

6 $$Y \, = \beta_{0} + \mathop \sum \limits_{i = 1}^{6} \beta_{1i} SO_{i} + \mathop \sum \limits_{j = 1}^{5} \beta_{2j} HR_{j} + \mathop \sum \limits_{k = 1}^{32} \beta_{3k} DS_{w} + \varepsilon .$$

Many other factors are also used for crops growth e.g. seed, watering, pesticides, land quality etc. which significantly impact the rice yield. This study incorporates these available factors in our dataset to accurately estimate the impact of climate variation on rice yield however the coefficients of sowing and harvesting time periods will remain in focused and the complete model will be

7 $$Y \, = \beta_{0} + \mathop \sum \limits_{i = 1}^{6} \beta_{1i} SO_{i} + \mathop \sum \limits_{j = 1}^{5} \beta_{2j} HR_{j} + \mathop \sum \limits_{k = 1}^{32} \beta_{3k} DS_{k} + \mathop \sum \limits_{n = 1}^{16} \beta_{41n } VR_{n} + \mathop \sum \limits_{n = 1}^{3} \beta_{42n} SC_{n} + \mathop \sum \limits_{n = 1}^{2} \beta_{43n} ST_{n} + \mathop \sum \limits_{n = 1}^{3} \beta_{44n } IM_{n} + \mathop \sum \limits_{n = 1}^{4} \beta_{45n} PM_{n} + \mathop \sum \limits_{n = 1}^{2} \beta_{46n} SMd_{n} + \mathop \sum \limits_{n = 1}^{2} \beta_{47n} SMc_{n} + \mathop \sum \limits_{n = 1}^{4} \beta_{48n} HM_{n} + \mathop \sum \limits_{n = 1}^{7} \beta_{49n} CR_{n} + \mathop \sum \limits_{n = 1}^{2} \beta_{410n } AA_{n} + \mathop \sum \limits_{n = 1}^{2} \beta_{411n} AP_{n} + \mathop \sum \limits_{n = 1}^{2} \beta_{412n} AW_{n} + \mathop \sum \limits_{n = 1}^{4} \beta_{413n} SL_{n} + \beta_{51} SQ + \beta_{52} MA + \beta_{53} UR + \beta_{54} DP + \beta_{55} SP + \beta_{56} SW + \beta_{57} WT + \beta_{58} OF + \varepsilon ,$$

where *VR* Crop varieties, *SC* Seed certification, *ST* Seed treatment, *IM* Irrigation mode, *PM* Plough machine, *SMd* Sowing mode, *SMc* Sowing machines, *HM* Harvesting machine, *CR* Crop rotation, *AA* Attack animal, *AP* Attack pest, *AW* Attack weed, *SL* Soil type, *SQ* Seed quantity, *UR* Urea, *DP* Diammonium Phosphate (DAP), *SP* Spray pest no., *SW* Spray weed, *WT* Watering no., *OF* Fertilizer other, *MA* Manure.

8 $$Y \, = \beta_{0} + \mathop \sum \limits_{i = 1}^{6} \beta_{1i} SO_{i} + \mathop \sum \limits_{j = 1}^{5} \beta_{2j} HR_{j} + \mathop \sum \limits_{k = 1}^{32} \beta_{3k} DS_{k} + \mathop \sum \limits_{m = 1}^{13} \mathop \sum \limits_{n = 1}^{16} \beta_{{{\text{4mn}}}} IP_{mn} + \mathop \sum \limits_{w = 1}^{8} \beta_{{{\text{5w}}}} X_{w} + \varepsilon ,$$

where

9 $$\mathop \sum \limits_{m = 1}^{13} \mathop \sum \limits_{n = 1}^{16} \beta_{4mn} IP_{mn} = \mathop \sum \limits_{n = 1}^{16} \beta_{41n} VR_{n} + \mathop \sum \limits_{n = 1}^{3} \beta_{42n} SC_{n} + \mathop \sum \limits_{n = 1}^{2} \beta_{43n} ST_{n} + \mathop \sum \limits_{n = 1}^{3} \beta_{44n} IM_{n} + \mathop \sum \limits_{n = 1}^{4} \beta_{45n } PM_{n} + \mathop \sum \limits_{n = 1}^{2} \beta_{46n} SMd_{n} + \mathop \sum \limits_{n = 1}^{2} \beta_{47n } SMc_{n} + \mathop \sum \limits_{n = 1}^{4} \beta_{48n} HM_{n} + \mathop \sum \limits_{n = 1}^{7} \beta_{49n } CR_{n} + \mathop \sum \limits_{n = 1}^{2} \beta_{410n} AA_{n} + \mathop \sum \limits_{n = 1}^{2} \beta_{411n} AP_{n} + \mathop \sum \limits_{n = 1}^{2} \beta_{412n} AW_{n} + \mathop \sum \limits_{n = 1}^{4} \beta_{413n } SL_{n} ,$$

and

10 $$\mathop \sum \limits_{w = 1}^{8} \beta_{5w} X_{w} = \beta_{51} SQ \, + \, \beta_{52} MA \, + \, \beta_{53} UR + \, \beta_{54} DP \, + \, \beta_{55} SP \, + \, \beta_{56} SW \, + \, \beta_{57} WT + \, \beta_{58} OF.$$

## Results and discussion

Regression results considering the pool data are estimated below to probe the impact of sowing and harvesting periods on rice yield.

Table 3 elucidates that the current sowing and harvesting time scales negatively influenced the rice yield. Based on 13,617 observations from 32 districts of the Punjab, estimated model reveals 14.4 percent fluctuation in yield with 15.64 kg/plot (20 × 15ft) germinal output. On average, postponing sowing and cutting for one-time scale may curtail the yield by 0.345 and 0.956 kg/plot (above 170 kg/acre), respectively. Variance inflation factor (VIF) value 1.33 expresses the absence of problematic multicollinearity.

**Table 3:** Sowing and harvesting impact on rice yield in the Punjab.

Variables	1
Sowing	− 0.345***
Harvesting	− 0.956***
Constant	15.64***
Observations	13,617
R-square	0.144

***p < 0.01, **p < 0.05, *p < 0.1, Robust standard errors.

Postponing sowing and harvesting by one the time period is estimated to reduce yield above 170 kg/acre which is above than 20% of average rice yield (PBS 2021). This average decline in yield is for one the time period delay in sowing and harvesting collectively however further delay may react viciously especially where temperature frequently crosses 40 °C in months of June and July Fig. S1 explains this reality. Fahad et al.^60^ investigated the plant growth and found rice development is extremely sensitive to temperature above 35 °C and predicted about 40% reduction in rice yield at the end of the twenty-first century due to climate variation. In this catastrophic scenario, finding the righteous time for rice sowing and harvesting seems helpful to reduce the climatic impact which is being investigated.

The model (in Table 3) explained the impact of sowing and harvesting scales (as a whole) while it is incapable to evaluate the individual sowing or harvesting time repercussions for finding suitable the climate of rice yield. Prearranged data description section describes the division of rice sowing and cutting time periods. These pre-owned time periods are used to re-evaluate the relationships of climate variability for rice yield to estimate the comparative ranking of suitable sowing and harvesting time periods.

Table 4 illustrates the influence of sowing and harvesting time periods on rice yield. The constant terms captured the impact of mentioned reference categosries based on 13,617 observations. R-square is the coefficient of determination and dots represent the omitted variables.

**Table 4:** Sowing and harvesting time impact on rice yield in the Punjab.

Variables	1	2	3^a^	4	5^a^	6^a^
**Sowing time**	–	–	–	–	–	–
Up to May 31	Ref.	2.15***	2.15***	.	.	11.39***
June 1–15	− 0.68***	1.47***	1.47***	12.06***	11.92***	10.71***
June 16–30	− 0.90***	1.25***	1.25***	11.19***	11.02***	10.49***
July 1–15	− 1.02***	1.14***	1.13***	10.86***	10.67***	10.38***
July 16–31	− 1.34***	0.81***	0.81***	10.50***	10.29***	10.05***
August onward	− 2.15***	Ref.	Ref.	9.66***	9.45***	9.24***
**Harvesting time**	–	–	–	–	–	–
Up to Sep. 30	Ref.	3.38***	3.37***	.	.	3.37***
Oct. 1–15	− 1.35***	2.03***	2.03***	1.55***	1.73***	2.03***
Oct. 16–31	− 2.25***	1.13***	1.12***	0.66***	0.86***	1.12***
Nov. 1–15	− 3.38***	Ref.	Ref.	− 0.42***	− 0.22	.
Nov. 16–30	− 3.31***	0.07	.	− 0.39*	.	.
Constant	14.77***	9.24***	9.24***	.	.	.
Observations	13,617	13,617	13,617	13,617	13,617	13,617
R-square	0.15	0.15	0.15	0.91	0.91	0.94

***p < 0.01, *p < 0.1.

^a^Robust standard errors.

Model 1 estimates the rice yield of 1969.33 kg/acre for crop sown and harvested in reference time periods (i.e. sown up to May 31 and harvested up to September 30). Deviation in cutting time from this reference group the rice yield may decline by, 179.33 kg/acre if harvested in the first half of October, 300.40 kg/acre if harvested in the second half of October, 450.40 kg acre if cutting in the first half of November and 441.33 kg if harvested in the second half of November. Deviation in sowing time from the reference/base group the rice yield may decline by, 90.53 kg/acre if sown in the first half of June, 120.13 kg/acre if sown in the second half of June, 135.48 kg/acre if sown in the first half of July, 178.67 kg/acre if sown in the second half of July and 286.93 kg/acre if sown from “August onward”.

Models 1 to 3 are estimated with a constant term which explained about 15 percent fluctuation in yield of rice and models from 4 to 6 are estimated without constant term and explained above 90 percent variation in the yield. Coefficients of sowing time periods captured the impact of the constant term (mentioned in previous models 1–3) and both techniques (with and without constant estimation) deliver almost similar outcomes. Results estimated yet, conveys that utilization of climate variation in the early rice season produces more yield compare with cultivating rice in a later season. It is suggested that climate variations during the early season for rice sowing and harvesting are suitable for rice yield and among these time slots sowing “Up to May 31” and harvesting “Up to Sep. 30” is preferable. The Later slots may reduce the rice yield.

To avoid the problem of heterocedasticity the robust standard errors (heterocedastic corrected) are calculated for models 3, 5 and 6. Low values of R-square in cross-sectional data and presence of multicollinearity in the categorical variable is logically understandable. As multiple factors may contribute to the output of a variable and these factors may change their behavior directly or indirectly due to additional factors in various cross sections thus the value of R-square which explained the variation in dependent variable due to selected independent variables may low. And the multicollinearity may be present in the categorical data as the presence of mutual characteristics is recognized by numerical values in a similar fashion.

Crops grow by utilizing various inputs and these factors bear important action and reactions for yield^62^. Utilization of seeds, fertilizer, pesticides, watering, crop rotation etc. alters the rice yield^70–72^ and utilization of inputs during improper growth stage reduce crops yield^73^. Merely, considering the climate variation a single source that impacts the rice yield may be exaggerating this impact and the investigation may lead toward biased outcomes. Resultantly, confounded relationship may be developed due to extraneous variables^74^ and the predictive capacity of the model may reduce which can be observed in the above table. To investigate for more reliable results by considering available factors to avoid the said drawbacks re-estimation is presented in Table 5.

**Table 5:** Rice yield as dependent variable in districts of the Punjab.

Variables	Coefficients	Variables	Coefficients
**Sowing times**		**Harvesting times**	
Up to May 31	0.574***	Up to Sep. 30	Reference
June 1–15	0.877***	Oct. 1–15	− 0.518***
June 16–31	0.853***	Oct. 16–31	− 1.061***
July 1–15	0.854***	Nov. 1–15	− 1.431***
July 16–31	0.627***	Nov. 16–30	− 1.411***
August onward	Reference	Observations	13,617
Constant	16.53***	R-Square	0.381

***p < 0.01, Robust standard errors.

Table S1 explains the variation in rice yield/plot with the usage of multiple factors show the estimated result for the rest of the factors. Reference category is imperative for proceedings of categorical variables as all the analyses may refer with base category. Changes in the reference category will alter the interpretations only. To avoid the problem of heterocedasticity, results are estimated using robust standard errors using software STATA and residual shows normality in Fig. S2.

In harvesting time other categories of this group are compared with its reference. Cutting during “October 1–15” is estimated to reduce 0.518 units less yield than the base group. All the coefficients in cutting times are negative which means the base group is most preferable and “November 1–15” is the least preferable cutting time. For smooth interpretation characteristics with expected extreme effects are taken as the reference categories. The sowing time periods are positive which shows that the base category is the least preferable. Including the rest of the factors (Table S1) in the estimated model altered the yield optimal sowing time period and now the sowing during “June 1–15” is the most preferable instead of “Up to May 31”. Rehman et al.^73^ suggested that inputs productive capacity changes with growth stages. Our results endorsed it while suggesting the compatibility of the sowing time period “June 1–15” with current input factors for optimal rice yield and validated Phan et al.^75^ which emphasized the need for correction of interfiled crop calendar. Abbas and Mayo^66^ found that increasing temperature during the replantation stage negatively affects rice plants by reducing the number of plants. Tu et al.^54^ tested six sowing dates to identifying the rice optimal sowing date for improvement and stabilizing the yield and estimated that utilizing proper sowing dates may increase 2–17 percent increase in rice yield. The estimated results of our study also match with Ding et al.^70^ which suggested earlier sowing than current dates to avoid flowering period on extremely hot days. Climate variation declines crop output through growth mismatching climatic factors^47, 67–69^. Our results are incompatible with Oad et al.^76^ which suggested the postponement of rice sowing to 15–30 days. This study addresses rice cultivation in Larkana district, Sindh, Pakistan and the results differ because of addressing different geographical region, climate change scenario or variation in practices during rice growth.

## Climate-induced vulnerability ranking

Yet the study investigated rice optimal sowing and harvesting time periods based on districts footprints of Punjab. Whether these climatic factors carry the same impact across the districts is unaddressed yet. The first possibility is the rice yield may remain the same across the districts with the usage of the same climate variation and secondly, the climate variation may behave differently and impact crystalizes through variation in district rice yield. To test the impact, an input is kept fixed to be utilized across all districts and district yield is tested against nonzero equal output and results are presented below.

Table 6 explains the inequality of rice yield across the districts by utilizing the same sowing and harvesting time periods. The variation in climatic impact in the districts of Punjab leads to estimate the district climate vulnerabilities for rice yield.

**Table 6:** Tests for equality of climate impact on rice across the districts.

Characteristics	F-test	Probability
**Sowing time**		
1	F(9, 237) = 4.69	Prob > F = 0.0000
2	F(22, 741) = 5.51	Prob > F = 0.0000
3	F(26, 1844) = 7.14	Prob > F = 0.0000
4	F(28, 5094) = 16.35	Prob > F = 0.0000
5	F(29, 4509) = 14.96	Prob > F = 0.0000
6	F(27, 769) = 3.96	Prob > F = 0.0000
**Harvesting time**		
1	F(18, 629) = 4.91	Prob > F = 0.0000
2	F(22, 2026) = 7.21	Prob > F = 0.0000
3	F(29, 4216) = 11.09	Prob > F = 0.0000
4	F(29, 5913) = 18.12	Prob > F = 0.0000
5	F(25, 469) = 6.47	Prob > F = 0.0000

The vulnerability of sowing and harvesting time periods are calculated and interpreted with reference to the base category. The observations of given districts are multiplied with estimated sowing and harvesting coefficients. Sowing and harvesting vulnerabilities are sum up to gain the district climate vulnerability value. Table S2 presents the district rice climate vulnerability ranking. The net climate loss or gain for each district is calculated in (kg/plot) and the districts have been ranked from more vulnerable to least vulnerable.

**Figure 4:**
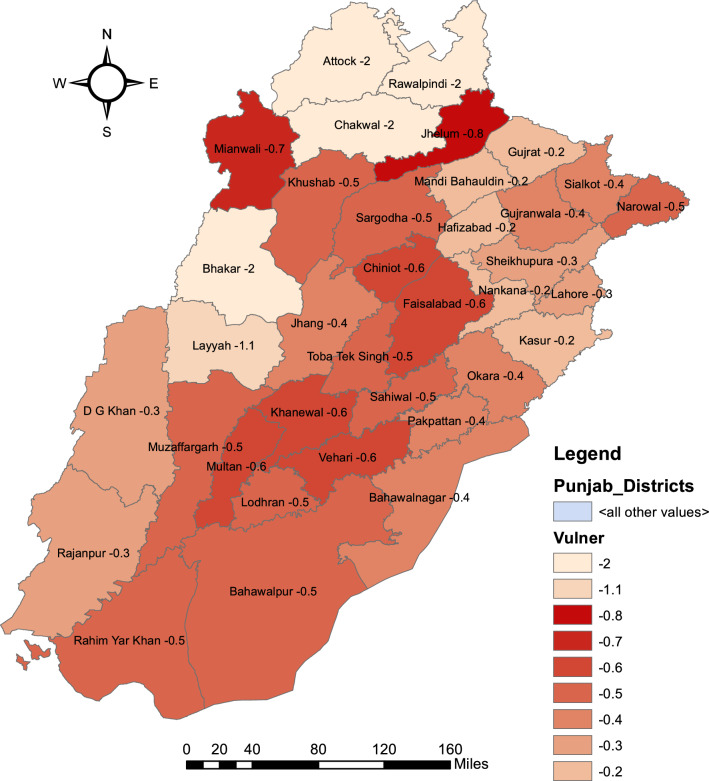
Spatial mapping of climate induced district vulnerability ranking for rice yield. *Source* Generating using Arc GIS version 10.3.1.

Figure 4 shows the hypothetical value of “− 2” for four districts to differentiate in spatial mapping, where reportedly, rice is not cultivated. The geographical region makes a semi-circle consists of Gujrat, Mandi Bahuddin, Hafizabad, Nankana and Kasur are the least climate vulnerable districts for rice crop in Punjab. Layyah, Jhelum and Mianwali are the most vulnerable districts and are scattered, the priority districts for adaptation measures for rice crop. The vulnerability pattern and its level may be seen for the rest of the districts from Fig. 4.

### Climate-induced yield and monetary loss ranking

Khan et al.^77^ suggested that rice farmers are more vulnerable in low yield regions than farmers in high yield regions of Punjab and regional priority must be given despite the difference in farm performance to reduce loss. The study in hand explores it and suggests the ranking of districts with greater farm-level loss, district rice yield loss and district monetary loss. District climate vulnerability ranking reports the district per unit adverse climate impact whereas the area under cultivation of rice crop varies in districts. Enumeration of cumulative district rice yield and monetary loss may crystalize the adverse climatic impact to emphasize the emergent circumstances for the implementation of adaptation strategies.

Table 7 explains the seasonal climate loss occurred to rice yield and its monetary value (Millions of PKR with 125/kg) in the districts of the province Punjab. It shows about 222.30 thousand metric tons of rice which is equal to 27.79 billion PKR climatic losses in single rice season because the usage of unadjusted climate rice season in the province of Punjab. District rice yield and monetary loss ranking differs significantly compared with district climate vulnerability ranking estimated in Table S2 because of differences in district rice cultivation area thus the district with small climate vulnerability shows the high monetary or rice yield loss due to considerable large area under rice cultivation or vice versa.

**Table 7:** Climate induced monetary and yield loss ranking of districts of the Punjab.

Ranking	Districts	Rice loss	Monetary loss	Ranking	Districts	Rice loss	Monetary loss
1	Gujranwala	34,920.24	4365.03	17	Khanewal	5021.45	627.68
2	Sialkot	19,487.42	2435.93	18	Vehari	4912.23	614.03
3	Sheikhupura	17,045.43	2130.68	19	Muzaffar Garh	4797.90	599.74
4	Okara	15,900.87	1987.61	20	Sahiwal	4135.44	516.93
5	Narowal	14,252.03	1781.50	21	Lahore	4013.45	501.68
6	Bahawalnagar	8977.41	1122.18	22	R. Y. Khan	3754.10	469.26
7	Hafizabad	8203.49	1025.44	23	Khushab	3698.86	462.36
8	Jhang	7324.22	915.53	24	D.G. Khan	3343.17	417.90
9	Sargodha	6876.93	859.62	25	Gujrat	3062.88	382.86
10	Nankana Sahib	6698.18	837.27	26	Layyah	2956.65	369.58
11	Chinniot	6650.93	831.37	27	Multan	2933.94	366.74
12	Pakpattan	6440.06	805.01	28	Bahawalpur	1397.46	174.68
13	M.B. Din	5731.24	716.41	29	Lodhran	1236.38	154.55
14	Kasur	5663.72	707.97	30	Mianwali	1052.68	131.59
15	Faisalabad	5595.33	699.42	31	Rajanpur	440.55	55.07
16	Tobatek Singh	5431.47	678.93	32	Jhelum	340.91	42.61

**Figure 5:**
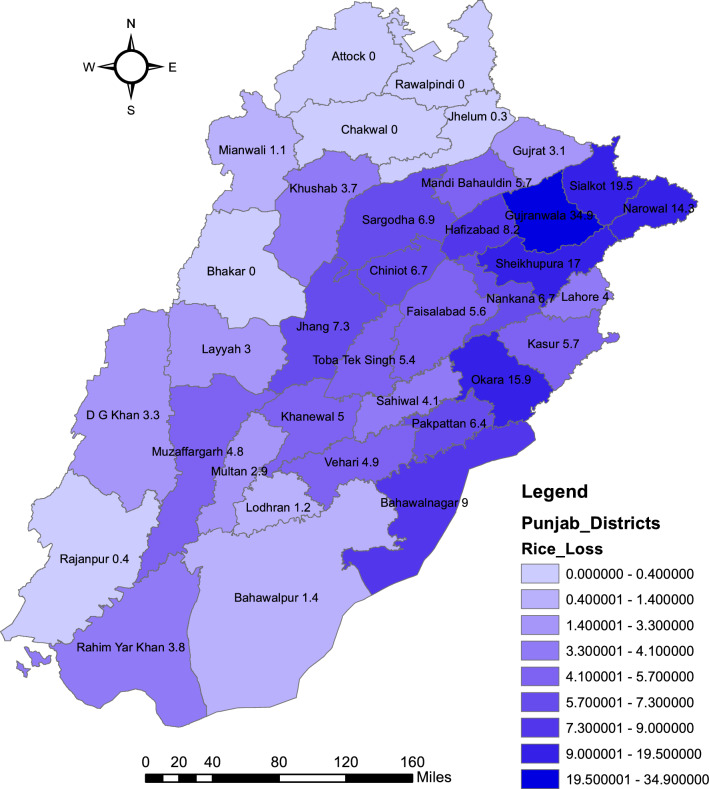
Spatial mapping of climate induced rice loss in districts of Punjab. *Source* Generating using Arc GIS version 10.3.1.

Figure 5 explains geo-mapping for district rice and monetary loss ranking. The districts less vulnerable to climate variations are facing more rice yield and ultimately monetary losses. The climate of central Punjab is considered most suitable for rice growth and the crop is cultivated in most of the land. Small vulnerability level in larger areas brings larger yield/monetary loss. Thus our results endorsed^77^ also suggest that low yield areas are more vulnerable to climate whereas deviating by contributing in literature by suggesting that the districts facing more yield/monetary losses may differ from the districts more vulnerable.

## Conclusions

Comprehensive dataset from the agriculture department of Punjab is obtained and 13,617 sampling units consist of categorical and numerical variables are formulated to use in this study. In addition to climate variation, the results are based on comprehensive model estimation including factors e.g. rice variety, seed quantity, seed treatment, watering times, fertilizer types, the quantity of fertilizers, use of pesticides, machinery use during sowing and harvesting, etc. which use during rice growth. Climate variability has negative repercussions in Punjab while the influence differs for districts and crops. The yield declining wallop escalates by postponing the crops sowing and cutting time. Based on estimated results Rice preferably may, sowing during “June 1–15” and with a small decline till “July 1–15”, harvesting “Up to September 30” and a small differential in the month of November. The influence of climate variability differs across the districts and most to least vulnerable districts for rice crop have been ranked which also highlights that the same policy to abate the climate vulnerability threat in all districts is unsuitable. Rice climate vulnerability ranking of all rice-growing districts of Punjab has been assessed where Layyah, Jhelum and Mianwali, are the most vulnerable while Mandi Bhauddin, Nankana Sahib, and Hafizabad are the least vulnerable districts. Spatial mapping showing the pattern of district vulnerabilities and monetary loss. District climate induced rice yield/monetary loss is estimated where district Gujranwala is bearing the most. Provincial climate induced rice yield loss is estimated to be 222.30 thousand metric tons of rice that is equal to 27.79 billion PKR in a single rice season because of the climate unadjusted customary rice season. Utilization of early sowing and avoiding late harvesting is suggested while preferable sowing time which provides optimal rice yield is estimated during June 1–15 and harvesting in the “end of September”. Vulnerability and yield/monetary loss may vary with the cultivation of the vulnerable areas.

### Policy implications

- Early sowing and harvesting of rice crop is recommended for better crop yield. This may reserves more days for potential crop growth.
- District of climate induced vulnerability, yield and monetary losses rankings highlight the priority territories those may require attention.
- Domestic climate resistant and high yield varieties seeds are required to be discovered further for which more focus may be required for research work in agricultural sector.
- Properly publicize the climate variation, impact on the crops, yield & monetary losses of farmers and urging for required adaptations measures.

### Suggestions for future studies

- Biochemical and physiological aspects of the crop can be investigated.
- Individual crop varieties can be studied which may suggest the crop variety specific suitable climate aspects.
- Macroeconomic aspects like crop rotation and domestic needs may be consider for future studies.

## Supplementary Information

Supplementary Information.

## Data Availability

The data is available with authors upon request.

## References

[1] Intergovernmental Panel on Climate Change. *Global Warming of 1.5 °C: An IPCC Special Report on the Impacts of Global Warming of 1.5 °C Above Pre-industrial Levels and Related Global Greenhouse Gas Emission Pathways, in the Context of Strengthening the Global Response to the Threat of Climate Change, Sustainable Development, and Efforts to Eradicate Poverty* (Intergovernmental Panel on Climate Change, 2018).

[2] Pakistan Economic Survey (2018–2019). Ministry of Finance, Government of Pakistan.

[3] Climate Change Indicators (CCI). (Pakistan Meteorological Department, Government of Pakistan, 2009).

[4] Parry, M. L., Canziani, O. F., Palutikof, J. P., Van Der Linden, P. J. & Hanson, C. E. Impacts, adaptation and vulnerability. Contribution of working group II to the fourth assessment report of the intergovernmental panel on climate change (IPCC, 2007). (Cambridge University Press, Cambridge, UK, 2007).

[5] Field, C. B. *et al*. IPCC, 2014: Climate Change 2014: impacts, adaptation, and vulnerability. Part A: global and sectoral aspects. Contribution of Working Group II to the Fifth Assessment Report of the Intergovernmental Panel on Climate Change. (Cambridge University Press, Cambridge, UK and New York, 2014).

[6] Eckstein, D., Hutfils, M. L., Winges, M., & Index, G. C. R. Who suffers most from extreme weather events? Weather-related loss events in 2017 and 1998 to 2017. Global Climate Risk Index (GCRI, 2019). (Germanwatch, Bonn, 2019).

[7] National Inventory Submissions 2019, United Nations Framework Convention on Climate Change (UNFCCC). https://unfccc.int/process-and-meetings/transparency-and-reporting/reporting-and-review-under-the-convention/greenhouse-gas-inventories-annex-iparties/national-inventory-submissions-2019 (2019).

[8] Franco, E. G. The global risks report 2020. in *World Economic Forum* (2020).

[9] Almeselmani M, Deshmukh PS, Sairam RK, Kushwaha SR, Singh TP (2006). Protective role of antioxidant enzymes under high temperature stress. Plant Sci..

[10] Wang Y, Dang F, Zheng X, Zhong H (2019). Biochar amendment to further reduce methyl mercury accumulation in rice grown in selenium-amended paddy soil. J. Hazard. Mater..

[11] Xing Y, Wang J, Shaheen SM, Feng X, Chen Z, Zhang H, Rinklebe J (2019). Mitigation of mercury accumulation in rice using rice hull-derived biochar as soil amendment: A field investigation. J. Hazard. Mater..

[12] He LX, Chen YL, Zhang TT, Zheng AX, Cheng Y, Du P (2019). Effects of different temperature conditions on yield and physiological properties of rice (*Oryza sativa* L.). Appl. Ecol. Environ. Res..

[13] Ali S, Liu Y, Ishaq M, Shah T, Ilyas A, Din IU (2017). Climate change and its impact on the yield of major food crops: Evidence from Pakistan. Foods.

[14] Kobata T, Palta JA, Tanaka T, Ohnishi M, Maeda M, KoÇ M, Barutçular C (2018). Responses of grain filling in spring wheat and temperate-zone rice to temperature: Similarities and differences. Field Crop Res..

[15] Pakistan Bureau of Statistics (PBS). Government of Pakistan. http://www.amis.pk/Agristatistics/Data/HTML%20Final/Rice/Yeild.html (2021).

[16] Ahmad A, Ashfaq M, Rasul G, Wajid SA, Khaliq T, Rasul F, Rosenzweig C (2015). Impact of climate change on the rice–wheat cropping system of Pakistan. Handbook of Climate Change and Agroecosystems: The Agricultural Model Intercomparison and Improvement Project Integrated Crop and Economic Assessments, Part 2.

[17] Helga W, Julia L (2019). The World of Organic Agriculture (WOA). Statistics and Emerging Trends 2019.

[18] Food and Agriculture Organization of the United Nations (2017). The Future of Food and Agriculture-Trends and Challenges. Annual Report.

[19] Apata, T. G., Samuel, K. D., & Adeola, A. O. Analysis of climate change perception and adaptation among arable food crop farmers in South Western Nigeria. In *International Association of Agricultural Economists (IAAE). 2009 Conference, Beijing, China. No. 1005-2016-79140* (2009).

[20] World Bank Group (2013). Global Financial Development Report 2014: Financial Inclusion.

[21] Degani E, Leigh SG, Barber HM, Jones HE, Lukac M, Sutton P, Potts SG (2019). Crop rotations in a climate change scenario: Short-term effects of crop diversity on resilience and ecosystem service provision under drought. Agr. Ecosyst. Environ..

[22] Fandjinou K, Zhang KB, Folega F, Mukete B, Yang XH, Wala K, Akpagana K (2019). Analysis of climate variability and its relations to vegetation dynamics in Togo, Western Africa from 1984 to 2017. Appl. Ecol. Environ. Res..

[23] Dudu H, Çakmak EH (2018). Climate change and agriculture: An integrated approach to evaluate economy-wide effects for Turkey. Clim. Dev..

[24] Khanal U, Wilson C, Hoang VN, Lee BL (2019). Autonomous adaptations to climate change and rice productivity: A case study of the Tanahun district, Nepal. Clim. Dev..

[25] Gaupp F, Hall J, Mitchell D, Dadson S (2019). Increasing risks of multiple breadbasket failure under 1.5 and 2 °C global warming. Agric. Syst..

[26] Scott GJ, Petsakos A, Juarez H (2019). Climate change, food security, and future scenarios for potato production in India to 2030. Food Secur..

[27] Hasan MM, Alauddin M, Sarker MAR, Jakaria M, Alamgir M (2019). Climate sensitivity of wheat yield in Bangladesh: Implications for the United Nations sustainable development goals 2 and 6. Land Use Policy.

[28] Beukes PC, Romera AJ, Neal M, Mashlan K (2019). Performance of pasture-based dairy systems subject to economic, climatic and regulatory uncertainty. Agric. Syst..

[29] Brown PR, Afroz S, Chialue L, Chiranjeevi T, El S, Grünbühel CM, Sacklokham S (2019). Constraints to the capacity of smallholder farming households to adapt to climate change in South and Southeast Asia. Clim. Dev..

[30] Tesfaye A, Hansen J, Kassie GT, Radeny M, Solomon D (2019). Estimating the economic value of climate services for strengthening resilience of smallholder farmers to climate risks in Ethiopia: A choice experiment approach. Ecol. Econ..

[31] Sheikh, M. M., Manzoor, N., Adnan, M., Ashraf, J. & Khan, A. M. Climate profile and past climate changes in Pakistan. (Global Change Impact Studies Center (GCISC)-RR-01, 2009).

[32] Khan MA, Tahir A, Khurshid N, Ahmed M, Boughanmi H (2020). Economic effects of climate change-induced loss of agricultural production by 2050: A case study of Pakistan. Sustainability.

[33] Khan NA, Gao Q, Abid M (2020). Public institutions’ capacities regarding climate change adaptation and risk management support in agriculture: The case of Punjab province, Pakistan. Sci. Rep..

[34] Food and Agriculture Organization of the United Nations. The state of food security and nutrition in the world. Annual Report (FAO, 2018).

[35] Mahmood N, Arshad M, Kaechele H, Shahzad MF, Ullah A, Mueller K (2020). Fatalism, climate resiliency training and farmers’ adaptation responses: Implications for sustainable rain fed-wheat production in Pakistan. Sustainability.

[36] Mahmood N, Arshad M, Kächele H, Ullah A, Müller K (2020). Economic efficiency of rainfed wheat farmers under changing climate: Evidence from Pakistan. Environ. Sci. Pollut. Res..

[37] Shabbir G, Khaliq T, Ahmad A, Saqib M (2020). Assessing the climate change impacts and adaptation strategies for rice production in Punjab, Pakistan. Environ. Sci. Pollut. Res..

[38] Punjab Agricultural Policy (PAP). (Department of Agriculture, Government of Punjab, Pakistan, 2018).

[39] Ahmad B, Haider S, Bukhari SAA (2015). Regional precipitation response to regional warming in past and future climate. Pak. J. Meteorol..

[40] Siddiqui R, Samad G, Nasir M, Jalil HH (2012). The impact of climate change on major agricultural crops: Evidence from Punjab, Pakistan. Pak. Dev. Rev..

[41] Krishnan P, Ramakrishnan B, Reddy KR, Reddy VR (2011). High-temperature effects on rice growth, yield, and grain quality. Adv. Agron..

[42] Wu C, Cui K, Wang W, Li Q, Fahad S, Hu Q (2016). Heat-induced phytohormone changes are associated with disrupted early reproductive development and reduced yield in rice. Sci. Rep..

[43] Khanal U, Wilson C, Hoang VN, Lee B (2018). Farmers' adaptation to climate change, its determinants and impacts on rice yield in Nepal. Ecol. Econ..

[44] Liu Y, Chen Q, Ge Q, Dai J, Qin Y, Dai L (2018). Modelling the impacts of climate change and crop management on phenological trends of spring and winter wheat in China. Agric. For. Meteorol..

[45] Smith P, Olesen JE (2010). Synergies between the mitigation of, and adaptation to, climate change in agriculture. J. Agric. Sci..

[46] Abid M, Scheffran J, Schneider UA, Ashfaq MJESD (2015). Farmers' perceptions of and adaptation strategies to climate change and their determinants: The case of Punjab province, Pakistan. Earth Syst. Dyn..

[47] Naqvi SAA, Ashfaq M, Adil SA, Ahmed A (2017). Current agricultural production system of Punjab is vulnerable to climate change: Impact assessment. J. Agric. Res..

[48] Foreign Agricultural Service. *United States Department of Agriculture* (2021). https://apps.fas.usda.gov/psdonline/circulars/grain-rice.pdf. (Accessed 04 September 2021).

[49] Romer PM (1994). The origins of endogenous growth. J. Econ. Persp..

[50] Food and Agriculture Organization of the United Nations and World Water Council (2015). Towards’ a Water and Food Secure Future: Critical Perspectives for Policy-Makers.

[51] Rehman A, Chandio AA, Hussain I, Jingdong L (2019). Fertilizer consumption, water availability and credit distribution: Major factors affecting agricultural productivity in Pakistan. J. Saudi Soc. Agric. Sci..

[52] Saccon P (2018). Water for agriculture, irrigation management. Appl. Soil Ecol..

[53] Shahzad MF, Abdulai A (2020). Adaptation to extreme weather conditions and farm performance in rural Pakistan. Agric. Syst..

[54] Tu D, Jiang Y, Liu M, Zhang L, Chen L, Cai M (2020). Improvement and stabilization of rice production by delaying sowing date in irrigated rice system in central China. J. Sci. Food Agric..

[55] Ali S, Saeed A, Kiani RS, Muhammad S, Khan F, Babar R (2021). Future climatic changes, extreme events, related uncertainties, and policy recommendations in the Hindu Kush sub-regions of Pakistan. Theor. Appl. Climatol..

[56] Fahad S, Adnan M, Hassan S, Saud S, Hussain S, Wu C, Wang D, Hakeem KR, Alharby HF, Turan V, Khan MA, Huang J, Hasanuzzaman M, Fujita M, Nahar K, Biswas JK (2019). Rice responses and tolerance to high temperature. Advances in Rice Research for Abiotic Stress Tolerance.

[57] Fahad S, Hussain S, Saud S, Khan F, Hassan S, Nasim W (2016). Exogenously applied plant growth regulators affect heat-stressed rice pollens. J. Agron. Crop Sci..

[58] Fahad S, Hussain S, Saud S, Hassan S, Ihsan Z, Shah AN, Wu C, Yousaf M, Nasim W, Alharby H, Alghabari F, Huang J (2016). Exogenously applied plant growth regulators enhance the morphophysiological growth and yield of rice under high temperature. Front. Plant Sci..

[59] Fahad S, Hussain S, Saud S, Hassan S, Tanveer M, Ihsan MZ, Shah AN, Ullah A, Nasrullah KF, Ullah S, AlharbyH NW, Wu C, Huang J (2016). A combined application of biochar and phosphorus alleviates heat-induced adversities on physiological, agronomical and quality attributes of rice. Plant Physiol. Biochem..

[60] Fahad S, Ihsan MZ, Khaliq A, Daur I, Saud S, Alzamanan S (2018). Consequences of high temperature under changing climate optima for rice pollen characteristics-concepts and perspectives. Arch. Agron. Soil Sci..

[61] Fahad S, Sönmez O, Saud S, Wang D, Wu C, Adnan M, Turan V (2021). Footprints of Climate Variability on Plant Diversity: Plant Growth Regulators for Climate-Smart Agriculture.

[62] Fahad S, Sonmez O, Saud S, Wang D, Wu C, Adnan M, Turan V (2021). Footprints of Climate Variability on Plant Diversity: Climate Change and Plants: Biodiversity, Growth and Interactions.

[63] Fahad S, Sonmez O, Saud S, Wang D, Wu C, Adnan M, Turan V (2021). Footprints of Climate Variability on Plant Diversity: Developing Climate Resilient Crops: Improving Global Food Security and Safety.

[64] Gilani H, Goheer MA, Ahmad H, Hussain K (2020). Under predicted climate change: Distribution and ecological niche modelling of six native tree species in Gilgit-Baltistan, Pakistan. Ecol. Indic..

[65] Ijaz M, Goheer MA (2021). Emission profile of Pakistan’s agriculture: Past trends and future projections. Environ. Dev. Sustain..

[66] Abbas S, Mayo ZA (2021). Impact of temperature and rainfall on rice reduction in Punjab, Pakistan. Environ. Dev. Sustain..

[67] Ahmad MJ, Iqbal MA, Choi KS (2020). Climate-driven constraints in sustaining future wheat yield and water productivity. Agric. Water Manage..

[68] Amir S, Saqib Z, Khan MI, Khan MA, Bokhari SA, Zaman-ul-Haq M, Majid A (2020). Farmers' perceptions and adaptation practices to climate change in rain-fed area: A case study from district Chakwal, Pakistan. Pak. J. Agric. Sci..

[69] Aznar-Sánchez JA, Piquer-Rodríguez M, Velasco-Muñoz JF, Manzano-Agugliaro F (2019). Worldwide research trends on sustainable land use in agriculture. Land Use Policy.

[70] Ding Y, Wang W, Zhuang Q, Luo Y (2020). Adaptation of paddy rice in China to climate change: The effects of shifting sowing date on yield and irrigation water requirement. Agric. Water Manage..

[71] Sileshi GW, Jama B, Vanlauwe B, Negassa W, Harawa R, Kiwia A, Kimani D (2019). Nutrient use efficiency and crop yield response to the combined application of cattle manure and inorganic fertilizer in sub-Saharan Africa. Nutr. Cycl. Agroecosyst..

[72] Vial LK, Molesworth A, Lefroy RD (2020). Balancing rice and non-rice crops: Managing the risks from soil constraints in Mainland Southeast Asian rice systems. Field Crop Res..

[73] Rehman A, Jingdong L, Shahzad B, Chandio AA, Hussain I, Nabi G, Iqbal MS (2016). Economic perspectives of major field crops of Pakistan: An empirical study. Pac. Sci. Rev. B Hum. Soc. Sci..

[74] Kothari C (2017). Research Methodology: Methods and Techniques.

[75] Phan H, Le Toan T, Bouvet A, Nguyen LD, Pham Duy T, Zribi M (2018). Mapping of rice varieties and sowing date using X-band SAR data. Sensors.

[76] Oad VK, Dong X, Arfan M, Kumar V, Mohsin MS, Saad S (2020). Identification of shift in sowing and harvesting dates of rice crop (*Oryza sativa* L.) through remote sensing techniques: A case study of Larkana district. Sustainability.

[77] Khan NA, Gao Q, Abid M, Shah AA (2021). Mapping farmers’ vulnerability to climate change and its induced hazards: Evidence from the rice-growing zones of Punjab, Pakistan. Environ. Sci. Pollut. Res..

