# Supplementary material for: Identification of climate induced optimal rice yield and vulnerable districts rankings of the Punjab, Pakistan
Source: Sci Rep. 2021 Dec 3;11:23393. doi: 10.1038/s41598-021-02691-4 (PMC8642530; doi:10.1038/s41598-021-02691-4)
Supplement: Supplementary file 1 — Supplementary Information. [file 41598_2021_2691_MOESM1_ESM.docx]

**Appendix**


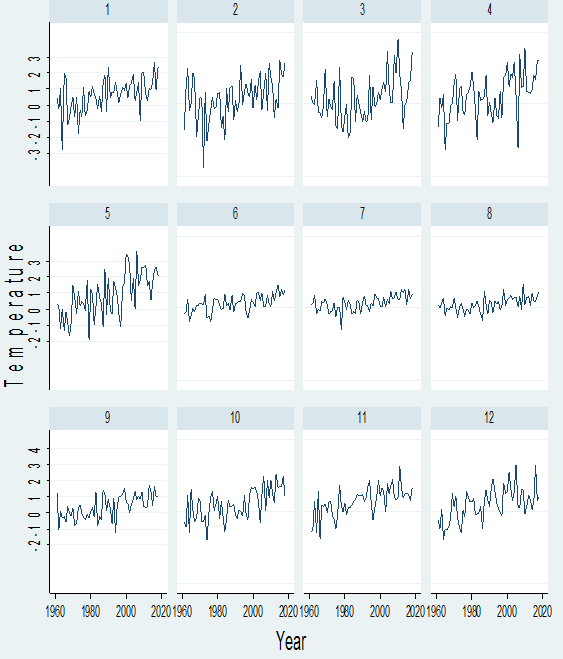


**Figure S1:** Temperature variability in Pakistan (1960-2018)

**3.1 Description of Variables**

**Yield** is the quantity of the crop from a plot of 15 x 20 square feet enumerated by agriculture department during round of surveys. This quantity can be multiplied with 133.3333 to calculate the yield per acre.

**District** represents the number of districts of the province Punjab, where the data collected for the crops. Reportedly, rice is sowing in 32 districts of the province Punjab.

**Sowing time** refers the start of the season for the crops. The crops are used to sow during this season. Here, the rice sowing season is divided in six sowing times.

**Harvesting time** refers the cultivation season of the crops. Rice harvesting season is divided in five harvesting times.

| **Figure S2:** Rice residual normality  **Table S1:** Rice yield as dependent variable in districts of the Punjab | | | | | | | | |  |  |
| --- | --- | --- | --- | --- | --- | --- | --- | --- | --- | --- |
| Variables | Coefficients | | Variables |  | Coefficients | | Variables Coefficients | | |  |
| Plough/Sowing Machines | | | Seed Characteristics | | | Districts | | |  |  |
| Chesel | | Reference | Seed treated | | 0.221*** | Bahawalpur | | -2.576*** |  |  |
| Disc | | -0.677*** | Seed quantity | | -0.0616* | Bahawalnagar | | -2.535*** |  |  |
| Manual | | -0.682*** | Soil Types | |  | Rah. y. Khan | | -2.915*** |  |  |
| Rota Weater | | -0.446*** | Chikny | | Reference | D. G. Khan 0.0282 | | |  | |
| Brdcast | | -0.0669 | Kalrathy | | -1.222*** | Jhelum | | Reference |  |  |
| Sow. Manual | | 0.0406 | Mayra | | 0.473*** | Sargodha | | -3.200*** |  |  |
| Rice varieties | | | Cutting Machines | | | Khushab | | -3.063*** |  |  |
| Bas: 385 | | -5.230*** | Com. Harvester | | Reference | Mianwali | | -3.681*** |  |  |
| Bas: 2000 | | -5.186*** | Thrasher | | -0.137 | Faisalabad | | -1.860*** |  |  |
| Bas: Kernel | | -4.369*** | Manual | | -0.212*** | Toba T. Singh | | -0.846 |  |  |
| Bas: Kinna | | -4.354*** | Last Crop | | | Jhang | | -1.929*** |  |  |
| Bas: Others | | -4.708*** | Fallow | | -0.886*** | Chinniot | | -2.978*** |  |  |
| Bas: Super | | | -4.880*** | | -0.932*** | Gujranwala | | -2.090*** |  |  |
| Irri: 6 | | -2.640*** | Maize | | Reference | Mandi B. D. | | -2.334*** |  |  |
| Irri: 9 | | -2.229*** | Others | | -0.984*** | Sialkot | | -1.280** |  |  |
| Irri: Fine | | -1.962*** | Wheat | | -1.046*** | Narowal | | -1.502*** |  |  |
| Irri: KS28 | | -1.993*** | Fertilizers | | | Gujrat | | -1.651*** |  |  |
| Irri: Others | | Reference | Oth. Fertilizers | | 0.00995*** | Hafizabad | | -1.759*** |  |  |
| Other: 386 | | -3.712*** | Dap | | 0.0218*** | Sheikhupura | | -2.703*** |  |  |
| Other: Indian | | -2.803*** | Urea | | 0.00965*** | NankanaSahib | | -3.393*** |  |  |
| Other: Others | | -3.573*** | Gobber | | 0.414*** | Lahore | | -2.826*** |  |  |
| Other: S_F | | -2.726*** | Spray pest No. | | 0.365*** | Kasur | | -3.587*** |  |  |
| Other: Sup | | -3.476*** | Spray weed No. | | 0.0963* | Okara | | -2.342*** |  |  |
| Risks/Diseases | | Reference | waste residual | | 0.0283*** | Sahiwal | | -2.307*** |  |  |
| attacked animal | | -0.764*** | Irrigation Modes | | | Pakpattan | | -2.395*** |  |  |
| attacked pest | | -0.0832 | Canal | | 0.263** | Multan | | -1.365*** |  |  |
| attacked weed | | -0.0633 | Mixed | | 0.239*** | Lodhran | | -2.598*** |  |  |
| Years | |  | Tube Well | | Reference | Khanewal | | -2.056*** |  |  |
| 2016 | | Reference | watering no. | | 0.0568*** | Vehari | | -2.219*** |  |  |
| 2017 0.0882* | | | Constant 16.53*** | | | Muzafargarh | | -2.995*** |  |  |
| 2018 | | 0.0139 | R-Square | | 0.381 | Layyah | | -1.594*** |  |  |
|  | |  | Observations | | 13,617 | Rajanpur | | -2.137*** |  |  |
| *** p<0.01, ** p<0.05, * p<0.1, Robust standard errors. | | | | | | | | |  |  |

| **Table S2:** Climate induced rice vulnerability ranking of districts of the Punjab | | | | | |
| --- | --- | --- | --- | --- | --- |
| Ranking | District | Vulnerability | Ranking | District | Vulnerability |
| 1 | Layyah | -1.0610 | 17 | Sahiwal | -0.4561 |
| 2 | Jhelum | -0.7990 | 18 | Gujranwala | -0.4421 |
| 3 | Mianwali | -0.6806 | 19 | Bahawalnagar | -0.4232 |
| 4 | Khanewal | -0.5959 | 20 | Sialkot | -0.3924 |
| 5 | Chinniot | -0.5785 | 21 | Pakpattan | -0.3843 |
| 6 | Multan | -0.5775 | 22 | Okara | -0.3726 |
| 7 | Faisalabad | -0.5749 | 23 | Jhang | -0.3600 |
| 8 | Vehari | -0.5524 | 24 | Lahore | -0.3488 |
| 9 | Narowal | -0.5240 | 25 | Rajanpur | -0.3239 |
| 10 | Khushab | -0.5205 | 26 | D.G. Khan | -0.2840 |
| 11 | Sargodha | -0.5194 | 27 | Sheikhupura | -0.2684 |
| 12 | R.Y. Khan | -0.5157 | 28 | Kasur | -0.2248 |
| 13 | Lodhran | -0.5152 | 29 | Gujrat | -0.2246 |
| 14 | Muzaffar Garh | -0.5061 | 30 | M. B. Din | -0.2240 |
| 15 | Bahawalpur | -0.4967 | 31 | Nankana Sahib | -0.1931 |
| 16 | Tobatek Singh | -0.4656 | 32 | Hafizabad | -0.1903 |
